# Armchair Janus WSSe Nanotube Designed with Selenium Vacancy as a Promising Photocatalyst for CO_2_ Reduction

**DOI:** 10.3390/molecules28124602

**Published:** 2023-06-07

**Authors:** Lin Ju, Xiao Tang, Jingli Li, Hao Dong, Shenbo Yang, Yajie Gao, Wenhao Liu

**Affiliations:** 1 School of Physics and Electric Engineering, Anyang Normal University, Anyang 455000, China; 201104015@stu.aynu.edu.cn (J.L.); 201101047@stu.aynu.edu.cn (Y.G.);; 2 Institute of Materials Physics and Chemistry, College of Science, Nanjing Forestry University, Nanjing 210037, China; xiaotang@njfu.edu.cn; 3 College of Physical Science and Technology, Central China Normal University, Wuhan 430079, China; dh123456@mails.ccnu.edu.cn; 4 Hongzhiwei Technology (Shanghai) Co., Ltd., 1599 Xinjinqiao Road, Pudong, Shanghai 200120, China; yangshenbo@hzwtech.com

**Keywords:** CO_2_ reduction, Se vacancy, photocatalysis, Janus WSSe nanotube

## Abstract

Photocatalytic conversion of carbon dioxide into chemical fuels offers a promising way to not only settle growing environmental problems but also provide a renewable energy source. In this study, through first-principles calculation, we found that the Se vacancy introduction can lead to the transition of physical-to-chemical CO_2_ adsorption on Janus WSSe nanotube. Se vacancies work at the adsorption site, which significantly improves the amount of transferred electrons at the interface, resulting in the enhanced electron orbital hybridization between adsorbents and substrates, and promising the high activity and selectivity for carbon dioxide reduction reaction (CO_2_RR). Under the condition of illumination, due to the adequate driving forces of photoexcited holes and electrons, oxygen generation reaction (OER) and CO_2_RR can occur spontaneously on the S and Se sides of the defective WSSe nanotube, respectively. The CO_2_ could be reduced into CH_4_, meanwhile, the O_2_ is produced by the water oxidation, which also provides the hydrogen and electron source for the CO_2_RR. Our finding reveals a candidate photocatalyst for obtaining efficient photocatalytic CO_2_ conversion.

## 1. Introduction

For the last few years, given the limitation of fossil fuel reserves and the growth of atmospheric CO_2_ levels, an urgent need has existed to create a sustainable option for converting unwanted CO_2_ into useful products in the form of chemicals and fuels [1,2,3], which will not only solve the greenhouse effect, melting glaciers, and other environmental problems caused by carbon dioxide, but also alleviate the current energy crisis [4]. The conversion of carbon dioxide could be operated through a variety of pathways, including biochemical [5], electrochemical [6,7], photochemical [8,9], and thermochemical [10] reactions. As sunlight is a theoretically unlimited power source, solar-powered CO_2_ reduction can be perceived as the best option among these promising approaches [11,12]. Until now, photocatalytic CO_2_RR has attracted great attentions and achieved many results [13,14,15,16]. Photocatalysis is widely believed to have three primary key steps, i.e., sunlight harvesting by the semiconductor (h*ν* > *E*g), photo-generated carrier separation and transport, and reactions on the surface [17,18,19,20]. While many solar active catalysts for CO_2_ photoreduction have been reported, they mostly suffer from instability, poor energy conversion rates, non-controllable selectivity, and failure to fully inhibit competing hydrogen evolution reactions (HER) in existence with water [21,22]. Consequently, it remains a great priority to design high-activity photocatalysts for CO_2_ reduction with great conversion efficiency and selectivity.

Soon after the Janus-structured MoSSe monolayer was fabricated by a modified chemical vapor deposition (CVD) method based on the sulfidation of MoSe_2_ monolayer [23] and the selenization of the MoS_2_ monolayer [24], the two-dimensional (2D) Janus transition metal dichalcogenides, such as Janus MoSSe and WSSe, have become candidates with great potential application for photocatalysis because of their excellent optical absorption, suitable band edge positions, and high carrier separation [19,23,24,25,26]. Our previous work demonstrated tha, the tubular Janus WSSe, obtained by rolling the planar Janus WSSe with an acceptable strain energy, possesses an enhanced electrostatic potential difference between the Se and S layers, resulting in a stronger built-in electric field than the planar structure. The stronger built-in electric field usually could help to strengthen the adsorption of small gas molecules, even to activate them. For CO_2_RR, due to the inertness of the CO_2_ gas molecule induced by the strong C=O bonds, effective activation of the CO_2_ molecule is key for the subsequent reduction. Therefore, to explore the photocatalytic CO_2_RR performance of the Janus WSSe nanotube is meaningful for developing a highly efficient photocatalyst. 

In our research, the CO_2_ adsorption on the Janus WSSe nanotube in pristine and defective states had been studied using DFT calculations. Adsorption energy ($E_{\mathrm{ads}}$), charge density difference (CDD), and density of state (DOS) were employed to explain the coupling between the substrate and adsorbate. It was found that the introduction of Se vacancies on Janus WSSe could brilliantly change the physical adsorption of CO_2_ into chemical adsorption, which effectively activates the CO_2_ gas molecules and makes CO_2_RR possible. The semiconducting property of the defective Janus WSSe nanotube is confirmed by the electronic band structure. We studied its photocatalytic CO_2_RR performance by analyzing the absorption spectrum, redox capacity, and reaction driving force of photo-excited carriers. Furthermore, in order to keep the CO_2_RR sustained and stable, we also consider the OER reaction on the S side of the defective Janus WSSe nanotube. Furthermore, competition from the CO_2_RR and hydrogen evolution reaction (HER) is addressed. We found that the defective WSSe nanotubes have excellent photocatalytic properties and can serve as a hopeful photocatalyst for light-driven CO_2_ reduction.

## 2. Results and Discussion

### 2.1. The CO_2_ Adsorption on Pristine Janus WSSe Nanotube

**Figure 1:**
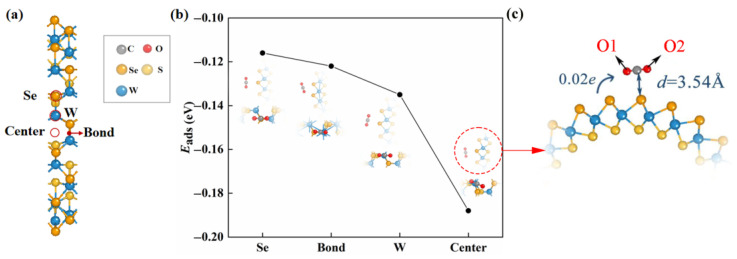
(**a**) The adsorption sites (marked with red circles) in consideration of the pristine Janus WSSe nanotube. (**b**) The adsorption energy as well as the top (upper) and side (lower) views of the optimized configurations of CO_2_ gas molecule adsorbing on pristine WSSe nanotube with different adsorption sites. The gray, red, orange, yellow, and blue balls each represent C, O, Se, S, and W atoms. (**c**) The enlarged view for the top view of center adsorption site optimized structure. The adsorption distance between the substrate and the adsorbate is represented by the dark blue, *d*. Transfer of charge from substrate to CO_2_ molecule identified by the blue arrow.

Janus WSSe nanotubes are constructed by scrolling Janus WSSe monolayers, whereby the W layer is interposed between the Se and S layers. Our previous work reported that the strain energy for the formation (0.10 eV/atom) of the Janus WSSe nanotubes with a structure of Se layer on the outside and S layer on the inside is lower than the one (0.23 eV/atom) with a contrary structure, indicating relatively more stability [27]. Herein, we chose the (12, 12) armchair Janus WSSe nanotube as the substrate in the adsorption system. As shown in Figure S1, the diameter is 21.86 Å and the height of Se-S is 3.22 Å. The W-S and W-Se bond lengths are 2.38 and 2.60 Å, respectively, separately a little shorter and larger than the corresponding ones (2.41 and 2.52 Å) in the planar structure [28]. In this study, we only considered the CO_2_ adsorption on the outer side (Se side) of the nanotube, and the case of the adsorption on the inner side is neglected because the CO_2_ gas molecules are difficult to pass through the nanotube walls to arrive on the inner side (the barrier is up to 28.33 eV, see Figure S2). We put a CO_2_ gas molecule on the Se side of the nanotube to build the adsorption system and completely relax it. As shown in Figure 1, there are four adsorption sites taken into consideration, namely **center** (above center of the hexagon), **bond** (above W-Se bond), and **W**/**Se** (above W/Se atom).

According to Equation (1), we obtained *E*_ads_ values of various adsorption sites, which were used to explore the most stable adsorption configuration. As shown in Figure 1b, we found that the *E*_ads_ arrived the smallest (−0.19 eV) when the adsorbed CO_2_ gas molecule was located at the **center** site, which was the most stable adsorption configuration. The small absolute value of *E*_ads_ of this adsorption configuration revealed that the adsorption is physical adsorption (usually, $\left|E_{\mathrm{ads}}\right|\leq 1$ eV [29,30,31,32]). 

We studied the mechanism of the CO_2_ physisorption on the pristine Janus WSSe nanotube in detail based on the adsorption distance and Bader charge results. The CO_2_ gas molecule kept the linear morphology after adsorption (see Figure 1c), and the distance from the C atom of the CO_2_ gas molecule to its nearest Se atom of the pristine Janus WSSe nanotube is as high as 3.54 Å, which greatly exceeds the Se-C bond length (2.29 Å). In addition, the amount of transfer electron, moving from the pristine Janus WSSe nanotube to the CO_2_ molecule, is only 0.02 *e*, indicating the weak interaction between the substrate and the CO_2_ molecule.

**Figure 2:**
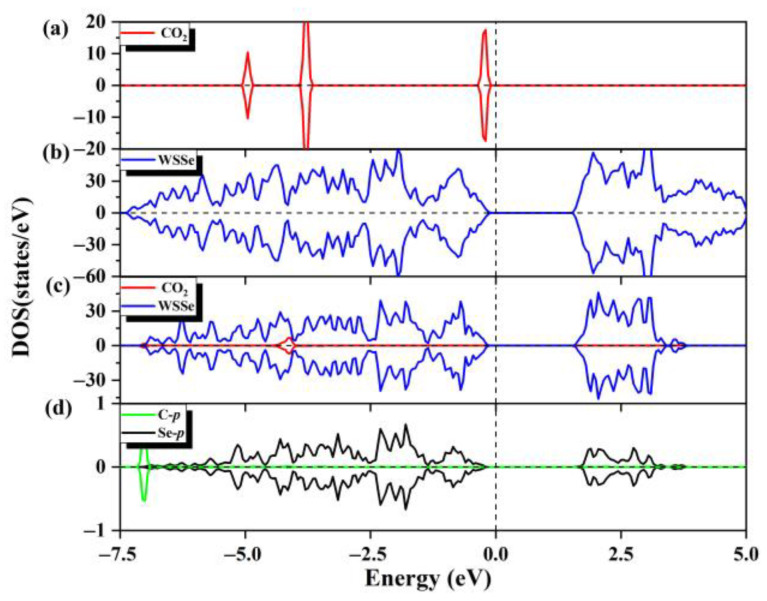
(**a**) The total state density of the pristine CO_2_ gas molecules and (**b**) the total state density of the pristine WSSe nanotube. (**c**) The partial state density of the adsorption system, where WSSe nanotube is shown in dark blue and CO_2_ is shown in red. (**d**) Partial state densities of the C *p* orbitals (cyan) of the adsorbed CO_2_ gas molecule and the Se *p* orbitals (black) of the Se atom most nearby the adsorbed CO_2_ molecule. Fermi level is expressed by the vertical dashed line.

At the same time, we also calculated the DOS values of the adsorption configurations. As can be seen from Figure 2a–c, the projected DOS of the WSSe nanotube has a negligible change compared with those of the corresponding pristine WSSe nanotube, indicating that the electronic properties of the WSSe nanotube remain. However, there is a significant difference of the DOS between the adsorbed gas molecule and the pristine gas molecule, which is due to charge rearrangement after adsorption; that is, the O atoms gain electrons, while the C atom loses electrons (as listed in Table S1). The little orbital hybridization between the WSSe nanotube and CO_2_, mainly composed of the Se *p* and CO_2_ O *p* orbitals, is consistent with the tiny interfacial electron transfer, demonstrating that the interaction between the WSSe nanotube and molecules is weak. According to the above analysis, it can be determined that the adsorption of CO_2_ by the pristine WSSe nanotube is physisorption.

### 2.2. The CO_2_ Adsorption on Defective Janus Wsse Nanotube

The pristine WSSe nanotube can be used as a gas collection system for physical CO_2_ adsorption. However, in order to convert the CO_2_ gas into value-added industrial raw materials through chemical reactions, chemical adsorption of CO_2_ is required, which requires the substrate to have a stronger adsorption capacity. Our earlier results have reported that introducing vacancy defects could effectively improve the stability of the geometric structures for some gas adsorption systems, making the adsorption capacity of the substrate increase [33,34,35].

**Figure 3:**
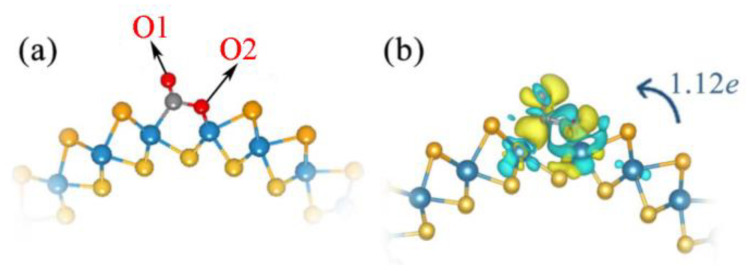
Top view (**a**) of the optimized structure and CDD (**b**) of Janus WSSe nanotube with Se vacancy adsorbed CO_2_ gas molecules. Cyan (yellow) areas indicate charge depletion (accumulation). The isosurface level is 0.002 *e*Å^−3^. Transfer of charge from substrate to CO_2_ molecule identified by the blue arrow.

Since the CO_2_ is more easily adsorbed on the Se side of WSSe nanotube, hereby, we applied the Se vacancy defects into the Janus WSSe nanotube to enhance its CO_2_ adsorption capacity, which also has been demonstrated to be more easily formed than the S and W vacancy defects in the WSSe layered material [33]. Based on the analysis on the elastic modulus, we find that a low Se vacancy concentration does not affect the mechanical property of the Janus WSSe nanotube drastically. (More details can be found in the Supporting Information and Figure S3). The calculated $E_{\mathrm{ads}}$ of CO_2_ molecule adsorbing on defective Janus WSSe nanotube is −1.41 eV, greatly exceeding the one (−0.19 eV) on the pristine Janus WSSe nanotube, indicating that the introduction of Se vacancy strengthens the CO_2_ adsorption. More interesting, as displayed in Figure 3a, the adsorbed CO_2_ molecule undergoes an obvious deformation from the initial linear shape into the bending one (∠OCO = 114.17°). Additionally, one of the C=O bonds in the adsorbed CO_2_ molecule (C-O_2_ bond) transforms into the C-O bond, and the C and O2 atoms bond to different W atoms, respectively. The obvious deformation demonstrates that the CO_2_ molecule could be activated by the defective Janus WSSe nanotube. However, the defective planar WSSe monolayer does not have such high activity. The adsorbed CO_2_ molecule on the defective planar WSSe monolayer keeps its linear shape (see Figure S4), and the adsorption energy in this case is only −0.20 eV. This phenomenon can be explained by the following reasons: (I) bending the planar structure allows more of the W atom area to be exposed, enlarging the contact surface of the CO_2_ molecule on the W atom; (II) the W atoms near the Se vacancy in the tubular structure WSSe have more electrons (0.15 *e*/atom) than the ones in the planar structure, according to the Bader charge results, which leads to easier electron transfer from W atoms on the WSSe nanotubes to the CO_2_ molecule and facilitates the formation of strong bonds. 

In the following, we further discuss the enhanced adsorption of CO_2_ on WSSe nanotubes with the introduction of Se vacancies, from the aspects of CDD, electron transfer, and DOS. As mentioned before, after CO_2_ adsorption at the Se vacancy site, C and O2 atoms separately bond to W atoms. As plotted in Figure 3b, the electron transfer amount from the defective Janus WSSe nanotube to the CO_2_ molecules is up to 1.12 *e*. The formation of C-W and O-W bonds indicate that on the defective Janus WSSe nanotube, the CO_2_ adsorption is chemical adsorption.

**Figure 4:**
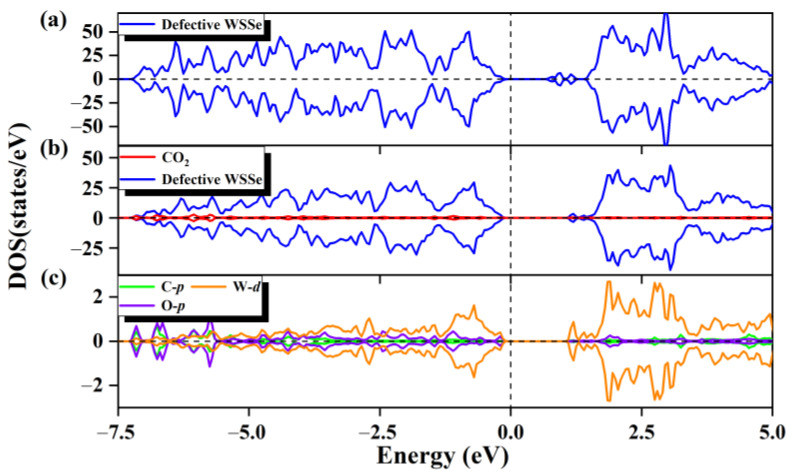
The total state density of WSSe nanotube with Se vacancy (**a**). The partial state density (**b**) of the adsorption system, the defective WSSe nanotubes are shown in dark blue, and CO_2_ is shown in red. (**c**) Partial state densities of adsorbed CO_2_ gas molecules in C *p* orbitals (cyan), O *p* orbitals (purple), and W *d* orbitals of two W atoms in substrate (orange). The vertical dashed line shows the Fermi level.

For the purpose of understanding the electronic origin of the chemisorption on the defective Janus WSSe nanotube, its corresponding DOS is calculated. For the defective Janus WSSe nanotube, its conduction band maximum (CBM) rises to a high level after the CO_2_ adsorption (see Figure 4a,b), which corresponds to the Bader charge result that the defective Janus WSSe nanotube loses 1.12 *e*. In addition, as shown in Figure 4c, there is an obvious orbital hybridization between the CO_2_ molecule and the defective Janus WSSe nanotube, which is mainly contributed by the O-*p* and C-*p* orbitals from the adsorbed molecule as well as the W-*d* orbitals from the W atoms in substrate bonding to the C and O2 atoms. This explains the phenomenon that the CO_2_ gas molecule is tightly attached to the defective Janus WSSe nanotube through the C-W and O-W bonds. In addition, the DOS of the CO_2_ molecules pre- and post-adsorption (see Figure 2a and Figure S5) shows that an obvious delocalization of DOS occurs after adsorption, which means a severe electron redistribution in the adsorbed CO_2_ gas molecule, caused by the gained electrons from the substrate. The results above provide more evidence that the adsorption of CO_2_ by the defective WSSe nanotubes is chemisorption. In other words, the introduction of Se vacancy can well convert the physical adsorption of CO_2_ into chemical adsorption on the Janus WSSe nanotube.

### 2.3. Photocatalytic Performance of Defective WSSe Nanotube for CO_2_RR

**Figure 5:**
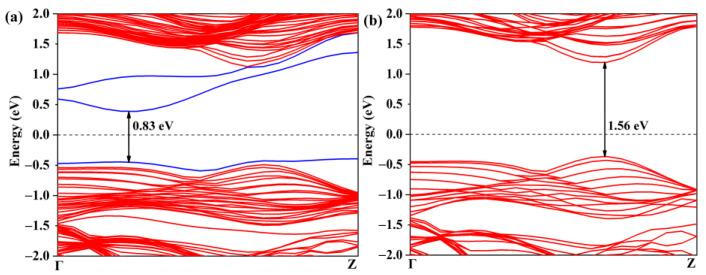
Band structures of (**a**) defective and (**b**) pristine Janus WSSe nanotubes. The gap states caused by the Se vacancy are marked with blue lines. The black number represents the value of band gap.

The activation of the CO_2_ gas molecule on the defective WSSe nanotube makes the further catalytic CO_2_ reduction reaction possible. As displayed in Figure 5a, though the Se vacancy bring about some gap states, the defective Janus WSSe nanotube still keeps the semiconductor character with a narrower band gap of 0.83 eV (the band gap of the pristine Janus WSSe nanotube is 1.56 eV, see Figure 5b). In the following, we studied the photocatalytic performance of the defective Janus WSSe nanotube.

**Figure 6:**
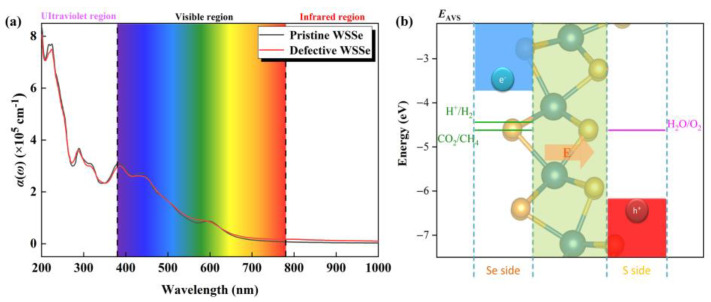
(**a**) Optical absorbance of pristine and defective Janus WSSe nanotubes. (**b**) Schematic diagram of band edge position of Janus WSSe nanotube relative to normal hydrogen electrode (NHE) at pH = 0. *E*_AVS_ represents the energy level relative to the absolute vacuum scale (AVS). The pink arrow represents the orientation of the built-in electric field.

In order to initiate the photocatalytic conversion of CO_2_, an efficient photocatalyst must have a high photo-conversion efficiency. As shown in Figure 6a, there are several significant light absorption peaks (over 10^5^ cm^−1^) among the visible light area for the pristine and defective Janus WSSe nanotubes, indicating they are promising catalyst candidates with visible-light responses. The highest absorption peak in the visible area for the pristine and defective Janus WSSe nanotubes arrive 3.10 × 10^5^ cm^−1^ (at 380.00 nm, black line) and 2.96 × 10^5^ cm^−1^ (at 380.00 nm, red line), which exceed the one of the planar Janus WSSe (1.30 × 10^5^ cm^−1^ at 466.28 nm) [34] and are on par with some reported photocatalysts, namely, MoSSe/graphene (4.00 × 10^5^ cm^−1^ at 500 nm) [36] and MoSSe/AlN (3.95 × 10^5^ cm^−1^ at 412 nm) [37]. Although the difference between the light absorption spectra of the pristine and defective Janus WSSe nanotubes are not significant, as displayed in Figure S6, in the infrared and visible regions, the optical absorption coefficient of the defective Janus WSSe nanotube is higher than the one of the pristine Janus WSSe nanotube, which is consistent with the fact that the defective Janus WSSe nanotube has a smaller band gap than the pristine one. The non-zero absorbance value in the infrared region (IR) of the defective WSSe nanotube ensures the utilization of IR photons. Therefore, the introduction of Se vacancy defects makes the Janus WSSe nanotube use photons in a relatively larger energy range. Additionally, the negligible difference of light absorption spectra between these two kinds of nanotubes may be caused by the fact that the gap states are too weak in the defective Janus WSSe, where the concentration of Se vacancy is too low (just 4.17%). In the visible region, the reported optical absorption coefficient of defective Janus WSSe monolayer with a higher concentration of Se vacancy (6.25%) is more obviously higher than the pristine Janus WSSe monolayer [34], which agrees well with the results of nanotubes.

In order for a semiconductor to be active for photo-reduction of CO_2_, the band edges must be aligned with the potentials of the reduction half-reactions [38]. On top of that, its band edge also needs to satisfy the oxidation potential of H_2_O/O_2_ because the oxygen evolution reaction (OER) could consume the redundant photo-excited holes and provide the necessary H^+^ + e^−^ pair simultaneously. As shown in Figure 6b, the CBM in the photocatalytic redox capacity is above the CO_2_/CH_4_ reduction potential, and the VBM is below the H_2_O/O_2_ oxidation potential, indicating that the WSSe nanotubes have sufficient redox capacity for both photocatalytic CO_2_RR and OER. Furthermore, our previous work pointed out that [27] the dipole caused by the structural asymmetry introduces a built-in electric field with the direction from the Se layer to the S layer (see the pink arrow in Figure 6b). In this case, the photoexcited electron and hole will run fast in opposite directions, causing high spatial separation of the electron–hole pairs, which surely suppresses the recombination of photoexcited carriers.

**Figure 7:**
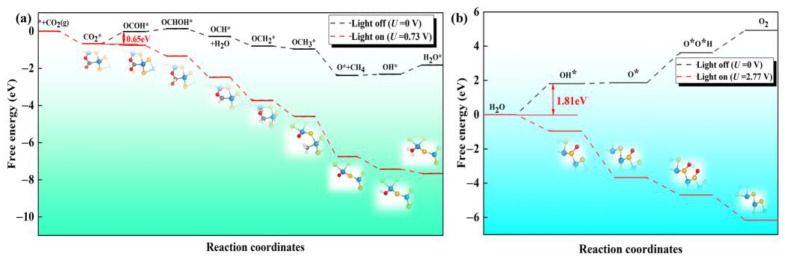
The Gibbs free energy diagrams for the (**a**) 8 *e* pathway of CO_2_RR and (**b**) 4 *e* pathway of OER on the defective Janus WSSe nanotube under different light conditions. The extra potentials provided by photogenerated electrons and holes are 0.73 and 2.77 V, respectively.

Next, we explore whether the reaction can be spontaneous under dynamic conditions. The case without any external potential (U = 0 V) is used to simulate the condition in darkness. We first screen the favorable reaction path of CO_2_RR on the defective Janus WSSe nanotube (see Figure S7). The CO_2_RR-to-CH_4_ process involves eight proton-coupled electron transfer steps (CO_2_ + 8H^+^ + 8e^−^ → 2H_2_O + CH_4_). The free energy diagram and the corresponding intermediates for the CO_2_RR-to-CH_4_ are shown in Figure 7a. The most possible path is CO_2_ * → OCOH * → OCHOH * → OCH * → OCH_2_ * → OCH_3_ * → O * →OH * → H_2_O *. The electrocatalytic steps, i.e., OCHOH * → OCH *, OCH * → OCH_2_ *, OCH_2_ * → OCH_3_ *, and OCH_3_ * → O *, are exothermic by −0.41, −0.51, −0.15, and −1.43 eV, respectively; meanwhile, the other hydrogenation steps, i.e., CO_2_ * → OCOH *, OCOH * → OCHOH *, O * → OH *, and OH * → H_2_O *, are endothermic by 0.65, 0.15, 0.06, and 0.50 eV, respectively. The formation of OCOH * is the potential determining step (PDS) with a limiting potential (*U*_l_) of −0.65 V. At the same time, we also investigated the OER process on the S side of the defective Janus WSSe nanotube along the 4 *e* transfer pathway, i.e., H_2_O → OH * → OOH * → O_2_ (see Figure 7b) [18,27]. The free energy changes (Δ*G*) for the four different steps are endothermic by 1.81, 0.06, 1.75, and 1.30 eV, respectively. The formation of OH * is the PDS with a *U*_l_ of −1.81 V.

According to the free energy calculations mentioned above, it could be found that both the CO_2_RR and OER have endothermic steps; thus, they could not take place spontaneously without photo-irradiation. However, the high enough external potential supplied by the photo-excited carriers helps to overcome the *U*_l_ of these redox half-reactions, making the redox half-reactions proceed spontaneously [39]. The extra potential of the photogenerated electrons/holes (*U*_e_/*U*_h_) is defined as the energy difference between H^+^/H_2_ reduction potential and the CBM/VBM [18,39,40,41]. According to our previous work [27], the *U*_e_ and *U*_h_ of the defective Janus WSSe nanotube at pH = 0 are 0.73 and 2.77 V, respectively, which are sufficient enough to separately cover the *U*_l_ of CO_2_RR and OER. Therefore, in consideration of *U*_e_ and *U*_h_, all the reduction and oxidation steps become downhill (red dash lines in Figure 7a,b). That is to say, under the light irradiation, both CO_2_RR and OER can operate spontaneously.

**Figure 8:**
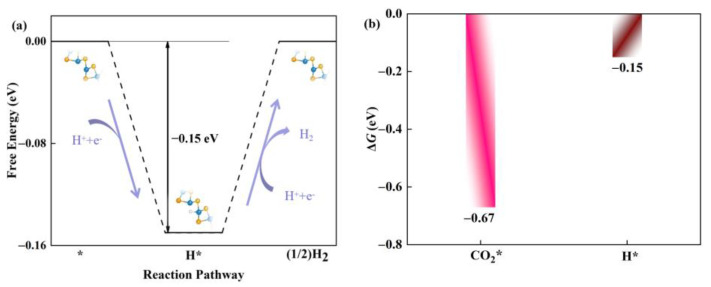
(**a**) Gibbs free energy diagram of HER on defective Janus WSSe nanotube. (**b**) Δ*G*co_2__*_ (pink bar) vs. Δ*G*_H*_ (brown bar) of defective Janus WSSe nanotube. * means the adsorption site.

Usually, the hydrogen evolution reaction (HER) is considered to be an important competitive side reaction in the catalytic CO_2_RR [42,43]. Next, we investigated the competitive relationship between CO_2_RR and HER in the defective Janus WSSe nanotubes. Based on the Brønsted-Evans-Polanyi relation [44,45], the reaction with lower Gibbs, Δ*G,* values has a smaller reaction barrier; thus, it is more favorable for kinetics. Accordingly, the Δ*G* for H * formation energy (Δ*G*_H*_) is calculated (Figure 8a) and compared with the one for CO_2_ * formation energy (Δ*G*_CO2*_). As shown in Figure 8b, Δ*G*_CO2*_ (−0.67 eV) is more negative than Δ*G*_H*_ (−0.15 eV), which ensures that the active sites are preferred to be occupied by CO_2_ *. Therefore, the defective Janus WSSe nanotube is more selective for CO_2_RR over HER.

## 3. Computational Methods

In our work, all the computational models are constructed with the DeviceStudio software [46]. In addition, the Geometric relaxation and electronic structure were conducted based on DFT simulations employing DS-PAW software [47]. The exchange-correlation energy of Perdew–Burke–Ernzerhof (PBE) was employed [48]. To depict the van der Waals (vdW) coupling in the adsorption system, we used the zero-damping DFT-D3 method suggested from Grimme [49]. All internal coordinates with fixed lattice constants were permitted to relax during the optimization process. The sampling integration of the Brillouin zone was performed in accordance with the Monkhorst-Pack scheme [50], and the structure optimization and electronic properties are calculated with a 1 × 1 × 4 K-point. The value of 500 eV was chosen as the cutoff energy of plane-wave basis. We set the periodic boundary condition along the z-axis and put more than 10.8 Å vacuum spaces along the *x* and *y* directions to avoid the interaction between adjacent nanotubes. Periodic boundary conditions were set on the z-axis and a vacuum space of more than 10.8 Å was applied on the x- and y-axes to evade adjacent nanotubes from interacting with each other. Moreover, the Δ*G* of CO_2_RR and OER were calculated using the computational hydrogen electrode (CHE) model [51]. Additional details of the Gibbs free energy simulations are available in the Supporting Information.

The $E_{\mathrm{ads}}$ of the CO_2_ on the WSSe nanotube was obtained from the following equation [52,53],

(1) $$E_{\mathrm{ads}}=E_{\mathrm{total}}-E_{\mathrm{sub}\;}-E_{{\mathrm{CO}}_{2}}\;$$

where $\;E_{\mathrm{total}}$ was the total energy of the adsorption system, while $E_{{\mathrm{CO}}_{2}}$ and $E_{\mathrm{sub}}$ separately were the total energies of the isolated CO_2_ molecule and the clean Janus WSSe nanotube. A higher negative $E_{\mathrm{ads}}$ indicated a more favorable exothermic adsorption. 

The plane-integrated CDD was carried out in accordance with the equation,

(2) $$\Delta \rho =\rho_{\mathrm{total}}-\rho_{\mathrm{sub}}-\rho_{{\mathrm{CO}}_{2}}$$

where $\rho_{\mathrm{total}}$, $\rho_{{\mathrm{CO}}_{2}}$, and $\rho_{\mathrm{sub}}$ separately were the charge density of the adsorption system, adsorbed CO_2_ molecule, and substrate.

The absorption coefficient, $a\left(\omega \right)$, used to estimate the solar energy gathering capacity was given by the following equation [27],

(3) $$a\left(\omega \right)=\sqrt{2}\frac{\omega }{c}{\left(\sqrt{\varepsilon_{1}{\left(\omega \right)}^{2}+\varepsilon_{2}{\left(\omega \right)}^{2}}-\varepsilon_{1}\left(\omega \right)\right)}^{\frac{1}{2}}\;$$

where *ε*_1_ and *ε*_2_ frequently were the real and imaginary parts of the frequency-dependent dielectric function, while *c* was the speed of light under vacuum.

## 4. Conclusions

In this paper, based on the first-principles calculations, we investigate the performance of the defective Janus WSSe nanotube for the photocatalytic CO_2_RR. The introduction of Se vacancy could significantly increase the amount of interfacial transferred electrons and lead to obvious electron orbital hybridization between adsorbates and substrates, making the CO_2_ adsorption on the Janus WSSe nanotube transform into chemisorption from physisorption. Strong chemisorption enables defective Janus WSSe nanotubes to be highly active and selective against CO_2_RR. In addition, the extra potential from photo-produced carriers is high enough to trigger spontaneous CO_2_RR and OER simultaneously on the defective Janus WSSe nanotube. For the first time, our work theoretically predicts the high photocatalytic performance of the defective Janus WSSe nanotube on CO_2_RR, which promisingly will stimulate extensive interests from material science and chemistry communities to realize our vision.

## Data Availability

The data presented in this study are available in Supplementary Materials.

## Supplementary Materials

The following supporting information can be downloaded at: https://www.mdpi.com/article/10.3390/molecules28124602/s1, Figure S1: Diameter, W-S bond length, W-S bond length and Se-S height of the pristine Janus WSSe nanotube; Figure S2: The relative adsorption energy and the location for the CO_2_ gas molecule passing through the pristine WSSe nanotube wall; Figure S3: Relative value of total energy variations as well as their corresponding fittings for the pristine and defective Janus WSSe nanotubes with respect to strain *ε* along the tube axis; Figure S4: Top view and side view of CO_2_ gas molecules adsorbed at the Se vacancy of Janus WSSe monolayer; Figure S5: The enlarged view for the partial density of states of CO_2_ portion from the adsorption system; Figure S6: The enlarged view the optical absorbance of pristine and defective Janus WSSe nanotubes at wavelength of 500–900 nm; Figure S7: The search process for the minimum energy reaction pathways of the CO_2_ reduction reactions on defective Janus WSSe nanotube; Table S1: The amount of charge transfer for C and O atoms of CO_2_ gas molecules adsorbed in pristine and defective Janus WSSe nanotubes, respectively; Free energy difference in the CO2RR and OER; Table S2: Zero-pint energy correction and entropy contribution of molecules and adsorbates in this study. Refs. [51,54] are cited in Supplementary Materials.
