# Supplementary material for: Armchair Janus WSSe Nanotube Designed with Selenium Vacancy as a Promising Photocatalyst for CO2 Reduction
Source: Molecules. 2023 Jun 7;28(12):4602. doi: 10.3390/molecules28124602 (PMC10302939; doi:10.3390/molecules28124602)
Supplement: Supplementary file 1 [file molecules-28-04602-s001.zip › molecules-2425694-supplementary.pdf]

Armchair Janus WSSe Nanotube Designed with Selenium Vacancy as a Promising Photocatalyst for CO_2 Reduction

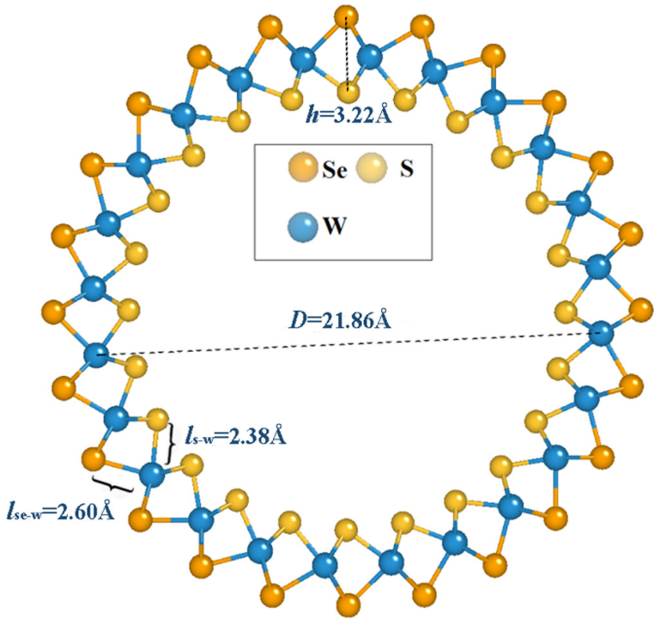

Figure S1. Diameter, W-S bond length ,W-S bond length and Se-S height of the pristine Janus WSSe nanotube.

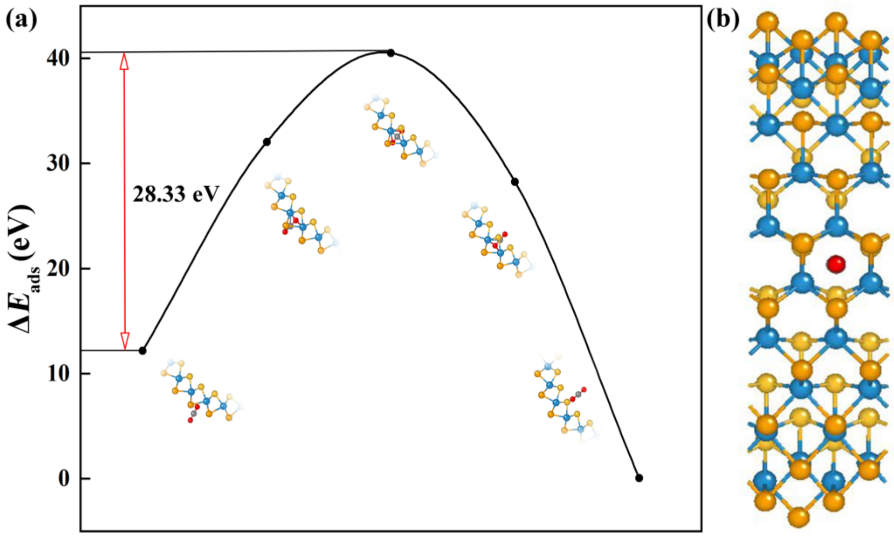

Figure S2. (a) The relative adsorption energy of the CO_2 gas molecule passing through the pristine WSSe nanotube wall from the center of the hexagonal lattice. (b) The location for the CO_2 gas molecule passing through the pristine WSSe nanotube wall.

The elastic modulus of pristine and defective Janus WSSe nanotubes

The elastic modulus C is calculated according to the following formula (equation S1):[1]

$$C = \left[\frac{\partial^2 E}{\partial \varepsilon^2}\right] / a_0 \qquad \text{(S1)}$$

where, a_0 is the lattice constant of Janus WSSe nanotube along the Z axis without strain, the strain ε is defined as $\varepsilon = \frac{a - a_0}{a_0}$, and E is the relative total energy of Janus WSSe nanotube under different strains (-0.03~0.03 along the Z axis), where the one without strain is treated as the standard value (seeing figure S3). The calculated elastic modulus of pristine and defective Janus WSSe nanotubes is 612.32 and 599.16 eV $Å^{-1}$, respectively. That is to say, the elastic modulus of Janus WSSe nanotube reduces merely 2.15% after the introduction of Se vacancy. Therefore, with a low Se vacancy concentration (4.17%), the mechanical property of Janus WSSe nanotube remains.

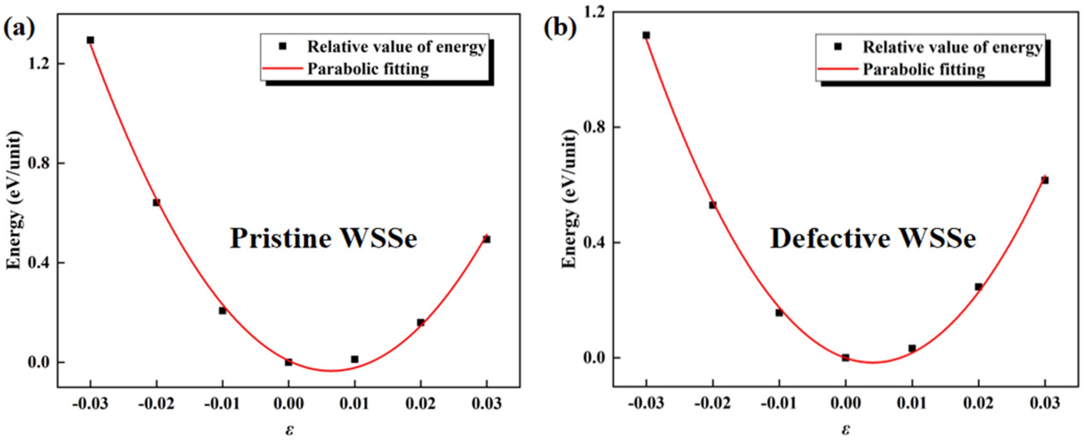

Figure S3. Relative value of total energy variations as well as their corresponding fittings for the pristine (a) and defective (b) Janus WSSe nanotubes with respect to strain ε along the tube axis.

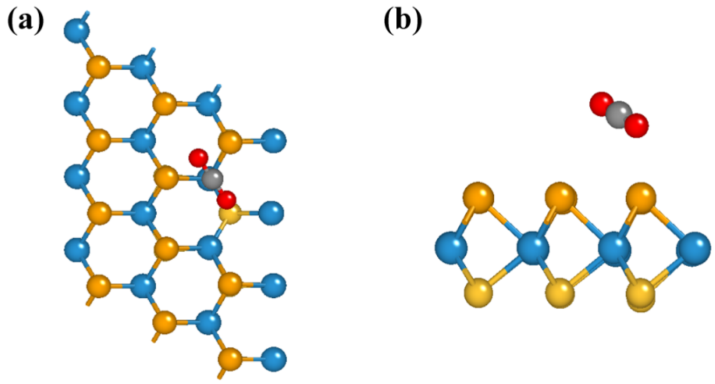

Figure S4. Top view (a) and side view (b) of CO_2 gas molecules adsorbed at the Se vacancy of Janus WSSe monolayer.

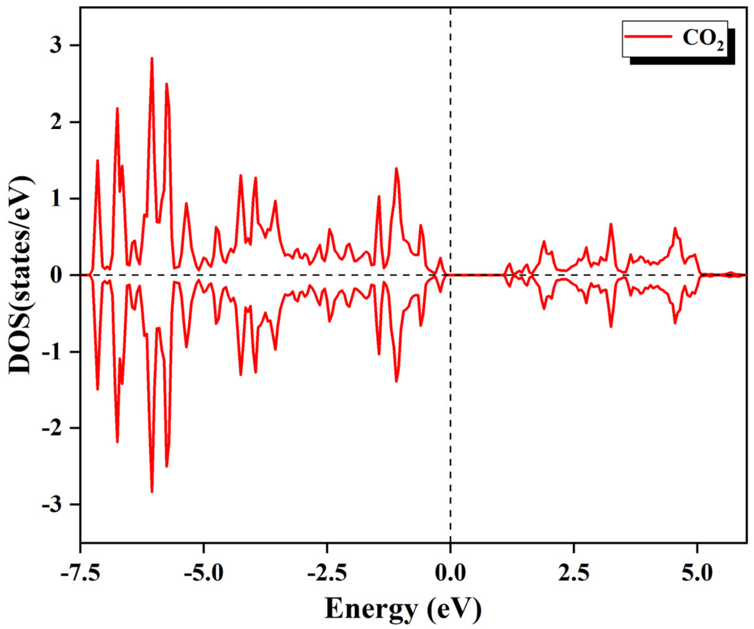

Figure S5. The enlarged view for the partial density of states of CO_2 portion from the adsorption system (CO_2 gas molecule adsorbed on defective Janus WSSe nanotube).

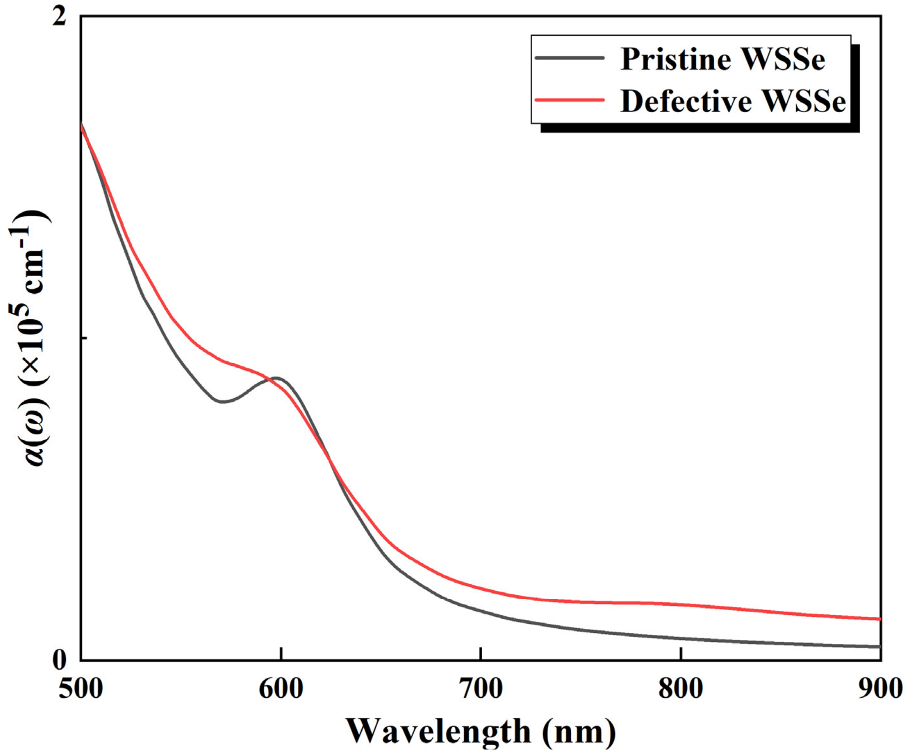

Figure S6. The enlarged view the optical absorbance of pristine and defective Janus WSSe nanotubes at wavelength of 500-900 nm.

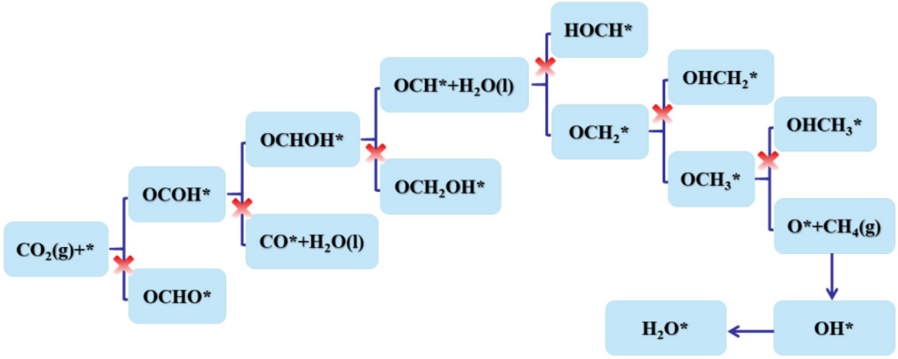

Figure S7. The search process for the minimum energy reaction pathways of the CO_2 reduction reactions on defective Janus WSSe nanotube. The red crosses denote disallowed reaction paths.

Table S1. The amount of charge transfer (in *e*) for C and O atoms of CO_2 gas molecules adsorbed in pristine and defective Janus WSSe nanotubes, respectively.

Sample	Atom		
	C	O1	O2
Pristine	-0.08	+0.05	+0.05
Defective	+1.06	+0.06	0.00

Free energy difference in the CO_2RR and OER

Free energy difference (ΔG) in the water redox reactions is calculated according the approach proposed by Nørskovet et al. [2] The formula at pH = 0 without solar irradiation can be defined as below (equation S2):

$$\Delta G = \Delta E + \Delta E_{\mathrm{zpe}} - T\Delta S \tag{S2}$$

where ΔE is the adsorption energy, ΔE_{zpe} and ΔS are the difference in zero point energy and entropy difference between the adsorbed state and the gas phase, respectively. Tables S2 presents E_{zpe} and TS (at 298.15 K) of the free molecules and the adsorbed species along the most favourable reaction pathway for CO_2-to-CH_4 reduction reaction happened on defective Janus WSSe nanotube.

There are eight steps to transform CO_2 into CH_4 molecule in the CO_2 reduction reaction (CO_2RR) along the minimum energy path, which can be written as:

$$CO_2^* + H^+ + e^- \rightarrow OCOH^* \tag{Sa}$$

$$OCOH^* + H^+ + e^- \rightarrow OCHOH^* \tag{Sb}$$

$$OCHOH^* + H^+ + e^- \rightarrow OCH^* + H_2O \tag{Sc}$$

$$OCH^* + H^+ + e^- \rightarrow OCH_2^* \tag{Sd}$$

$$OCH_2^* + H^+ + e^- \rightarrow OCH_3^* \tag{Se}$$

$$OCH_3* + H^+ + e^- \rightarrow O* + CH_4 \quad (Sf)$$

$$O* + H^+ + e^- \rightarrow OH* \quad (Sg)$$

$$OH*+ H^+ + e^- \rightarrow H_2O* \quad (Sh)$$

where * means the adsorbed site, CO_2*, OCOH*, OCHOH*, OCH*, OCH_2*, OCH_3*, O*, OH* and H_2O* represent the adsorbed intermediates.

Meanwhile, hydrogen production half reaction can be decomposed into four steps, the reaction equation can be written as:

$$*+H_2O \rightarrow *OH + H^+ + e^- \quad (Si)$$

$$*OH \rightarrow *O + H^+ + e^- \quad (Sj)$$

$$*O + H_2O \rightarrow *O*OH + H^+ + e^- \quad (Sk)$$

$$*O*OH \rightarrow *O_2 + H^+ + e^- \quad (Sl)$$

For each reaction of both water oxidation and hydrogen production, the free energy difference under the effect of pH and an extra potential bias can be written as:

$$\Delta G_{\mathrm{Sa}} = \Delta G_{\mathrm{OCOH}*} = G_{\mathrm{OCOH}*} - \frac{1}{2} G_{\mathrm{H_2}} - G_{CO_2*} + \Delta G_U + \Delta G_{pH} \quad (S3)$$

$$\Delta G_{\mathrm{Sb}} = \Delta G_{\mathrm{OCHOH}*} = G_{\mathrm{OCHOH}*} - G_{\mathrm{OCOH}*} - \frac{1}{2} G_{\mathrm{H_2}} + \Delta G_U + \Delta G_{pH} \quad (S4)$$

$$\Delta G_{\mathrm{Sc}} = \Delta G_{\mathrm{OCH}*} = G_{\mathrm{OCH}*} + G_{H_2O} - \frac{1}{2} G_{H_2} - G_{\mathrm{OCHOH}*} + \Delta G_U + \Delta G_{pH} \quad (S5)$$

$$\Delta G_{\mathrm{Sd}} = \Delta G_{\mathrm{OCH_2}*} = G_{\mathrm{OCH_2}*} - G_{\mathrm{OCH}*} - \frac{1}{2} G_{H_2} + \Delta G_U + \Delta G_{pH} \quad (S6)$$

$$\Delta G_{\mathrm{Se}} = \Delta G_{\mathrm{OCH_3}*} = G_{\mathrm{OCH_3}*} - G_{\mathrm{OCH_2}*} - \frac{1}{2} G_{H_2} + \Delta G_U + \Delta G_{pH} \quad (S7)$$

$$\Delta G_{\mathrm{Sf}} = \Delta G_{\mathrm{O}*} = G_{\mathrm{O}*} + G_{CH_4} - G_{\mathrm{OCH_3}*} - \frac{1}{2} G_{H_2} + \Delta G_U + \Delta G_{pH} \quad (S8)$$

$$\Delta G_{\mathrm{Sg}} = \Delta G_{\mathrm{OH}*} = G_{\mathrm{OH}*} - G_{\mathrm{O}*} - \frac{1}{2} G_{H_2} + \Delta G_U + \Delta G_{pH} \quad (S9)$$

$$\Delta G_{\mathrm{Sh}} = \Delta G_{\mathrm{H_2}O*} = G_{\mathrm{H_2}O*} - G_{\mathrm{OH}*} - \frac{1}{2} G_{H_2} + \Delta G_U + \Delta G_{pH} \quad (S10)$$

$$\Delta G_{\mathrm{Si}} = \Delta G_{\mathrm{OH}*} = G_{\mathrm{OH}*} + \frac{1}{2} G_{H_2} - G_{\mathrm{H_2}O} + \Delta G_U + \Delta G_{pH} \quad (S11)$$

$$\Delta G_{\mathrm{Sj}} = \Delta G_{O*} = G_{O*} + \frac{1}{2} G_{H_2} - G_{\mathrm{OH}*} + \Delta G_U + \Delta G_{pH} \quad (S12)$$

$$\Delta G_{\mathrm{Sk}} = \Delta G_{*\mathrm{O}*\mathrm{OH}} = G_{*\mathrm{O}*\mathrm{OH}} + \frac{1}{2} G_{H_2} - G_{O*} - G_{\mathrm{H_2}O} + \Delta G_U + \Delta G_{pH} \quad (S13)$$

$$\Delta G_{\mathrm{Sl}} = \Delta G_{*} = G_{*} + G_{O_2} + \frac{1}{2} G_{H_2} - G_{*\mathrm{O}*\mathrm{OH}} + \Delta G_U + \Delta G_{pH} \quad (S14)$$

Where ΔG_{pH} ($\Delta G_{pH} = \mathrm{k_B} T \times ln10 \times pH$) represents the free energy contributed in different pH

concentration. ΔG_U ($\Delta G_U = -eU$) denotes extra potential bias provided by an electron in the electrode, where U is the electrode potential relative to the standard hydrogen electrode (SHE). The catalytic activity was evaluated by the theoretical limiting potentials, U_l (U_l = -ΔG_{max}/e), where ΔG_{max} is the free energy change of the most thermodynamically unfavorable elementary step, i.e., the potential-determining step (PDS).

Table S2. Zero-pint energy correction (E_{ZPE}) and entropy contribution (TS, T=298.15 K) of molecules and adsorbates in this study.

**Species**	E_{ZPE} **(eV)**	$-TS$ **(eV)**
H_2	0.28	-0.40
H_2O	0.56	-0.67
CO_2*	0.30	-0.14
OCOH*	0.62	-0.17
OCHOH*	0.94	-0.15
OCH*	0.50	-0.07
OCH_2*	0.82	-0.09
OCH_3*	1.04	-0.15
O*	0.08	-0.04
OH* (Se side)	0.38	-0.05
H_2O*	0.63	-0.08
OH* (S side)	0.36	-0.08
O*	0.08	-0.05
OOH*	0.45	-0.11

1. Li, X.; Dai, Y.; Ma, Y.; Li, M.; Yu, L.; Huang, B., Landscape of DNA-like inorganic metal free double helical semiconductors and potential applications in photocatalytic water splitting. *J. Mater. Chem. A* **2017,** 5, (18), 8484-8492.
2. Nørskov, J. K.; Rossmeisl, J.; Logadottir, A.; Lindqvist, L.; Kitchin, J. R.; Bligaard, T.; Jónsson, H., Origin of the Overpotential for Oxygen Reduction at a Fuel-Cell Cathode. *J. Phys. Chem. B* **2004,** 108, (46), 17886-17892.
